# Tracking Antigen-Specific T-Cells during Clinical Tolerance Induction in Humans

**DOI:** 10.1371/journal.pone.0011028

**Published:** 2010-06-09

**Authors:** Aamir Aslam, Hsien Chan, David A. Warrell, Siraj Misbah, Graham S. Ogg

**Affiliations:** 1 Medical Research Council Human Immunology Unit, National Institute of Health Research Biomedical Research Centre, Weatherall Institute of Molecular Medicine, University of Oxford, Oxford, United Kingdom; 2 Nuffield Department of Clinical Medicine, University of Oxford, Oxford, United Kingdom; 3 Department of Clinical Immunology, John Radcliffe Hospital, Oxford, United Kingdom; New York University, United States of America

## Abstract

Allergen immunotherapy presents an opportunity to define mechanisms of induction of clinical tolerance in humans. Significant progress has been made in our understanding of changes in T cell responses during immunotherapy, but existing work has largely been based on functional T cell assays. HLA-peptide-tetrameric complexes allow the tracking of antigen-specific T-cell populations based on the presence of specific T-cell receptors and when combined with functional assays allow a closer assessment of the potential roles of T-cell anergy and clonotype evolution. We sought to develop tools to facilitate tracking of antigen-specific T-cell populations during wasp-venom immunotherapy in people with wasp-venom allergy. We first defined dominant immunogenic regions within Ves v 5, a constituent of wasp venom that is known to represent a target antigen for T-cells. We next identified HLA-DRB1*1501 restricted epitopes and used HLA class II tetrameric complexes alongside cytokine responses to Ves v 5 to track T-cell responses during immunotherapy. In contrast to previous reports, we show that there was a significant initial induction of IL-4 producing antigen-specific T-cells within the first 3–5 weeks of immunotherapy which was followed by reduction of circulating effector antigen-specific T-cells despite escalation of wasp-venom dosage. However, there was sustained induction of IL-10-producing and FOXP3 positive antigen-specific T cells. We observed that these IL-10 producing cells could share a common precursor with IL-4-producing T cells specific for the same epitope. Clinical tolerance induction in humans is associated with dynamic changes in frequencies of antigen-specific T-cells, with a marked loss of IL-4-producing T-cells and the acquisition of IL-10-producing and FOXP3-positive antigen-specific CD4+ T-cells that can derive from a common shared precursor to pre-treatment effector T-cells. The development of new approaches to track antigen specific T-cell responses during immunotherapy can provide novel insights into mechanisms of tolerance induction in humans and identify new potential treatment targets.

## Introduction

The ability to induce clinical tolerance in humans carries enormous therapeutic potential. Whilst allergen desensitization is a long established approach for allergic disease [1]–[4], the critical underlying mechanisms are still debated. Early clinical tolerance occurs in the face of persisting antigen-specific IgE and can be mediated, at least in part, by the passive transfer of IgG derived from previously exposed, but tolerant individuals [4]. Thus specific IgG blocking antibodies have been implicated in the generation of early clinical non-responsiveness [5]–[6] and may be induced through local and systemic IL-10 derived from regulatory T cells and other cells [5]. Indeed CD4+CD25+ cells have been documented to accumulate systemically and at sites of allergen immunotherapy in some studies of successful desensitization [7]–[10]. Allergen desensitization may also be accompanied by a shift from a largely Th2 dominated cytokine production pattern to one in which Th1 cytokines predominate [11]–[44]. Whole antigen immunotherapy carries a risk of anaphylaxis, and therefore peptide based approaches have attracted significant interest. However peptide immunotherapy carries risk of late phase disease exacerbation, possibly due to induction of antigen-specific effector T cell responses [45]–[50]. Such exacerbations may be peptide dose dependent and clearly it will be important to define the optimal parameters for success [51].

Studies of T cell responses during immunotherapy have largely used approaches based on analysis of overall populations of cells or on cytokine-producing sub-populations of cells derived after antigenic stimulation. In contrast, HLA-peptide tetrameric complexes facilitate the identification and characterisation of antigen-specific T cells without the need for the cells to express particular functional activities. Such an approach therefore allows the analysis of populations of antigen-specific T cells at the epitope level and potentially permits a longitudinal assessment of the relative contributions of deletion, anergy, cytokine switching and regulatory T cell induction to the changing T cell profile during immunotherapy.

Wasp venom immunotherapy is licensed in the United Kingdom for the management of individuals with a history of severe systemic reactions to wasp stings. We have previously shown that the dominant antigens recognized by T cells are antigen 5 (Ves v 5), hyaluronidase (Ves v 2) and phospholipase A1 (Ves v 1) [52] which are coincident with the main IgE-binding components. In addition, a number of antigenic regions of antigen 5 have been described with differential recognition patterns between allergics and non-allergics [53]. However in order to apply the HLA-peptide tetrameric complex technology to the study of antigen-specific T cell responses, it is essential to have detailed epitope mapping data, which are not currently available for wasp venom specific proteins.

We sought to develop tools to enhance our ability to track antigen-specific T cells during immunotherapy. We were limited to using HLA class II restrictions for which HLA peptide tetrameric complexes could be generated. Based on previous studies, we focused on HLA-DRB1*1501 as this allele is relatively common within the UK and HLA-DRB1*1501-restricted responses specific to other allergens can be detectable *ex vivo* and after short term culture [54]–[56].

## Results

### Patient characteristics

We recruited 23 wasp allergic patients that had systemic allergic reactions. Table 1 details the severity of reaction according to Brown's grading [57], the proportion of males and females, the mean wasp venom specific IgE responses and the mean age. The HLA DR types are given in table 2.

**Table 1:** Clinical characteristics of the 23 patients enrolled in the study.

Sex	Male; number of patients (percentage)	16 (66%)
	Female; number of patients (percentage)	8 (34%)
Age (mean, standard deviation)		54 (11.04) years
Severity of anaphylaxis	Brown's grade 2; number of patients (percentage)	15 (63%)
	Brown's grade 3; number of patients (percentage)	9 (37%)
Wasp venom specific IgE, mean (standard deviation)		19 KU/L (10)

**Table 2:** HLA-DR types of 23 patients enrolled in the study.

HLA type	number of patients (percentage)
HLA DRB1*01	5 (21)
HLA DRB1*03	6 (25)
HLA DRB1*04	6 (25)
HLA DRB1*07	5 (21)
HLA DRB1*08	1 (4)
HLA DRB1*11	6 (25)
HLA DRB1*12	3 (13)
HLA DRB1*13	2 (8)
HLA DRB1*14	1 (4)
HLA DRB1*15	6 (25)
HLA DRB1*16	1 (4)

### Antigen 5-specific functional T cell responses

Having previously documented that high frequencies of wasp venom-specific T cells circulate in allergic individuals and that antigen 5 (Ves v 5) is a dominant target antigen [52], we proceeded to characterize constituent epitopes. Using peripheral blood mononuclear cells (PBMC) derived from 23 wasp allergic individuals, we first investigated the presence of circulating cytokine-producing T cells that were reactive to overlapping peptide pools derived from Ves v 5 (antigen 5, 4 pools of 20 amino acid peptides, each overlapping by 10 amino acids). The *ex vivo* frequency of IL-4 producing cells was low, with mean response of 12.4 spot forming units (sfu)/million PBMC for Ves v 5 and interestingly the majority were directed against peptide pool 4 (Figure S1). We therefore examined the proliferative capacity of the Ves v 5-specific T cells using cultured ELISpot. Following 10 days in culture, we observed significant expansion of Ves v 5-specific responses with a mean of 1032 sfu/million cells for IL-4 and 781.3 sfu/million cells for IFNγ (Figure S2). The IL-4 responses were significantly higher in individuals with allergic disease than in exposed non-allergic individuals (1032 sfu/million cells and 353.1 sfu/million cells respectively, P<0.05), but the IFNγ responses were not significantly different (781.3 sfu/million cells and 481.9 sfu/million cells respectively, p = 0.18).

**Figure 1:**
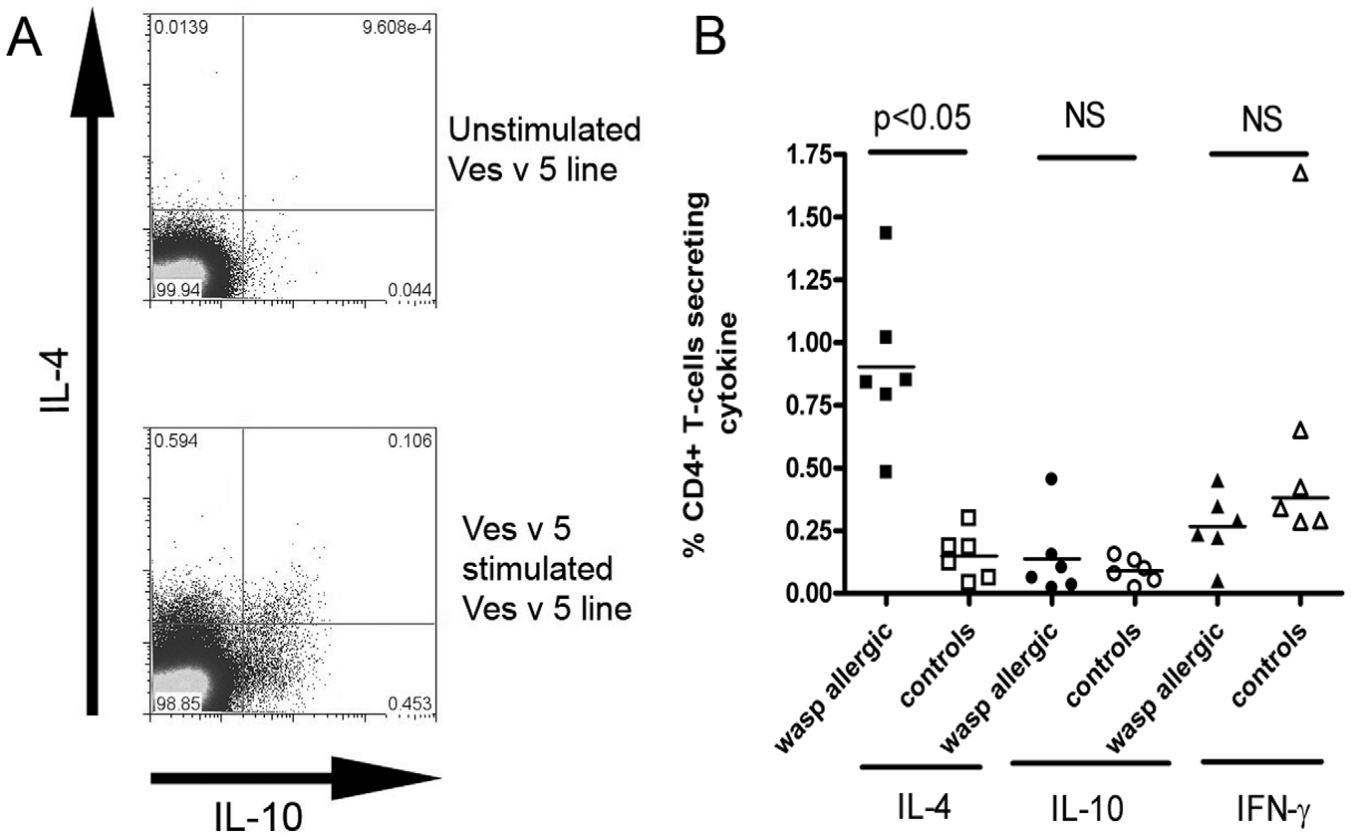
Ves v 5 specific cytokine responses in wasp allergy. A. T-cells were cultured for 10 days in the presence of recombinant Ves v 5 and IL-4 and IL-10 production was determined after re-stimulation with recombinant Ves v 5 (lower) and PBS - negative control (top). B. Cytokine responses of Ves v 5 T-cell lines after re-stimulation with antigen. IL-4 responses to Ves v 5 is significantly higher in individuals with a history of wasp venom anaphylaxis (N = 6) than non-allergic controls (N = 6) (P<0.05), whereas the frequency of IL-10 and IFN-γ responses does not differ between the 2 groups; student's t-test.

We also used whole recombinant Ves v 5 to confirm that the antigen is processed and presented, leading to proliferation in antigen-specific T cells. PBMC derived from allergic and non-allergic individuals were incubated with recombinant Ves v 5 (1 µg/ml), and then tested 10 days later for cytokine productivity in response to recombinant Ves v 5. Figure 1 shows examples of such analyses and that IL-4 producing T cells specific to recombinant Ves v 5 were significantly more common in allergic individuals than healthy controls. Interestingly, there was no significant difference in the proportion of Ves v 5-specific IL-10 and IFN-γ producing cells between the groups. Furthermore, although the cytokine production was largely contained within the CD4+ subset of cells, it was clear that allergen-specific CD8+ T cells were also detected after day 10 cultures (data not shown).

Overall these results showed that both wasp allergic and exposed non-allergic individuals have circulating Ves v 5-specific CD4+ T cells that are able to proliferate and produce effector cytokines, but in allergic individuals the frequencies of such cells are higher and the responses biased towards Th2 cytokine production.

### Identification of DRB1*1501-restricted epitopes within Ves v 5

**Figure 2:**
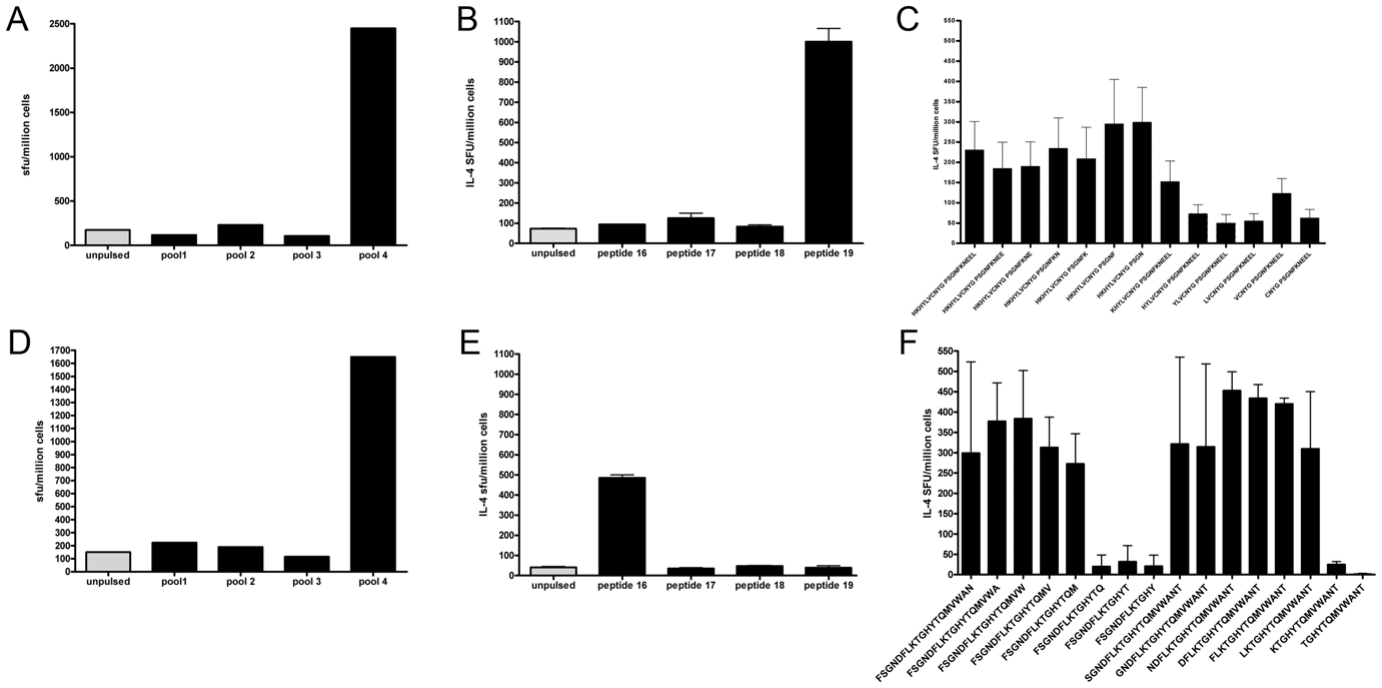
Identification of DRB1*1501-restricted epitopes within Ves v 5. T-cell lines from two wasp allergic patients to rVes v 5 were re-stimulated with Ves v 5 peptide pools (a and d) and IL-4 ELISpot responses were detected to pool 4. DRB1*1501 transfected L-cells were then separately pulsed with constituent peptides from peptide pool 4 and used to stimulate rVes V 5 lines; peptides 19 and 16 were thus identified as containing HLA-DRB1*1501 restricted epitopes (b and e, respectively). T-cell lines were generated against rVes v 5 from 5 venom allergic patients and IL-4 responses were determined for different p19 (c) and p16 (f) derived sequences to reveal the minimal epitopes. SFU = spot forming units.

Having characterised the overall Ves v 5-specific responses to recombinant protein and overlapping pools, we were able to proceed to map individual T cell epitopes. We were able to map DRB1*1501 restricted epitopes by first expanding allergen specific T-cells *in vitro* by incubating PBMC from DRB1*1501 allergic individuals with recombinant Ves v 5. These T-cell lines were then screened for cytokine production at day 10 of culture using DRB1*1501-transfected murine L cells pulsed with the Ves v 5-derived peptide pools. We noted that pool 4 frequently induced responses and therefore proceeded to define the reactive constituent peptides (Figure 2A and D). In some individuals, peptide 19 induced the DRB1*1501 restricted response (example in Figure 2B). The epitope was fine mapped using serial truncations from peptide termini and showed the optimal epitope to be HKHYLVCNYGPSGN (Figure 2C). In other individuals, peptide 16 induced the dominant DRB1*1501 restricted responses (Figure 2E). The minimal epitope was also fine mapped using serial truncations and found to be LKTGHYTQM (Figure 2F). However the optimal response was noted to comprise the slightly longer peptide NDFLKTGHYTQMVWA (data not shown). Furthermore, the NDFLKTGHYTQMVWA 15mer was predicted by an online class II prediction software syfpeithi (http://www.syfpeithi.de/).

**Figure 3:**
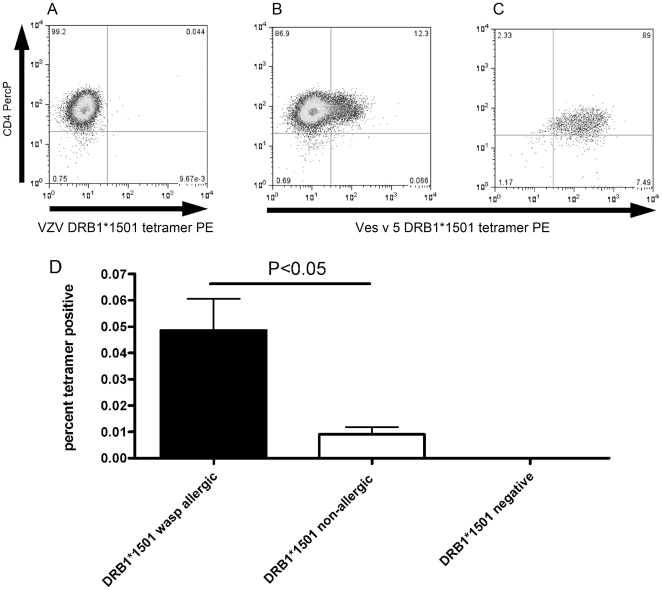
Detection of Ves v 5 specific T-cells with MHC Class II Ves v 5 tetrameric complexes. Examples of DRB1*1501 tetramer staining of a day 14 cultured population using a control DRB1*1501 Varicella zoster virus peptide tetramer (a) and a wasp venom Ves v 5 DRB1*1501 tetramer (b). The Ves v 5 DRB1*1501 tetramer was also used to stain a clonal population (c). d. Ex vivo frequency of tetramer-binding cells from individuals with a history of anaphylaxis to wasp venom (N = 6) was significantly higher than from DRB1*1501-positive non-allergic controls (with a history of wasp stings, N = 4) and DRB1*1501-negative individuals (N = 4); p<0.05, student's t-test.

Having mapped the novel epitopes within antigen 5, we were in a position to use Ves v 5/DRB1*1501 peptide tetrameric complexes. As well as staining *ex vivo*, we were also able to use tetramer-guided cell sorting to generate T cell clones from allergic individuals. Figure 3a-c shows examples of tetrameric complex staining of day 14 cultures and of a clonal population of cells; 3a shows staining of the Ves v 5 line to a control varicella zoster virus/DRB1*1501 tetramer. Figure 3d shows overall *ex vivo* responses in patients and controls.

### Longitudinal functional responses

**Figure 4:**
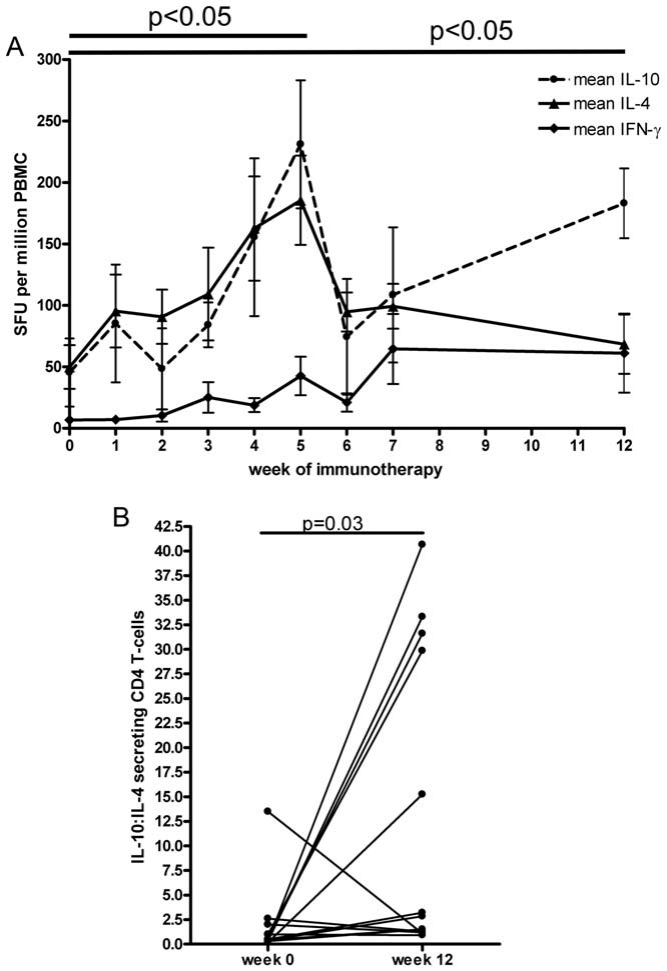
Longitudinal ex-vivo cytokine responses to immunotherapy. A. Ex vivo longitudinal frequencies of cytokine producing cells during immunotherapy in response to whole wasp venom in 13 wasp allergic patients. Circles IL-10, triangles IL-4, diamonds IFN-γ. SFU (spot forming units). There was a significant effect of the week of immunotherapy on the frequency of IL-10 and IL-4 secreting T-cells (one-way ANOVA p = 0.0284 and p = 0.0368, respectively) and there was a significant rise in IL-4 and IL-10 responses at week 5 (p<0.05), which for IL-10 was maintained at 12 weeks after starting immunotherapy (p<0.05). Means and SD are displayed. B. Ratio of IL-10:IL-4 secretion over time during immunotherapy in the same 13 wasp allergic patients; p = 0.03 student's paired t-test.

Previous studies have suggested that there is a progressive loss of IL-4 producing allergen-specific T cells during immunotherapy [15], [20]–[21], [38], [58]–[60]. However, these studies detect cytokine responses after in-vitro expansion of cells and few studies have examined frequent responses at weekly intervals during immunotherapy. By determining *ex vivo* frequencies of wasp venom specific cytokine secreting T-cells by ELISpot and in contrast to previous studies, we observed an initial significant (P<0.05) induction of IL-4 producing T cells specific for whole wasp venom that reached a peak between weeks 3–5 of immunotherapy before falling through to week 12 (Figure 4). Furthermore we observed a similar induction of IL-10 producing cells during weeks 3-5 but after a subsequent fall, this was followed by a sustained increase to week 12 (Figure 4). Figure 4b shows the IL-10 to IL-4 ratio at weeks 0 and 12 of immunotherapy. These longitudinal changes in frequency of cytokine secreting cells are also antigen-specific, as IFN-γ responses to a “FEC” control peptide pool (32 CD8+ T-cell epitopes from EBV, CMV, and influenza [61]) did not change over time (Figure S3). In addition to these changes in the frequency of antigen-specific cytokine secreting T-cells we also observed an increase in venom specific IgG4 during immunotherapy (Figure S4).

**Figure 5:**
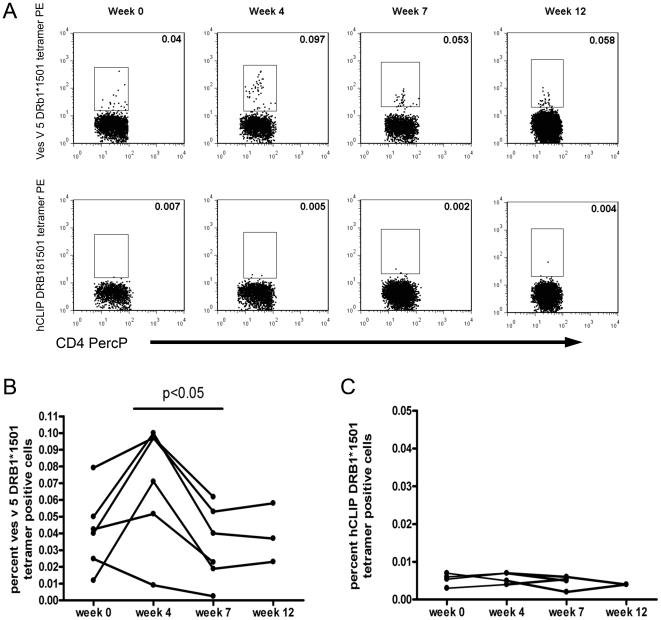
Longitudinal analysis of tetramer-binding cells during immunotherapy. A. The top line of dot-plots show the frequency of HLA-DRB1*1501 Ves V 5 peptide-tetramer binding cells from a wasp allergic patient before (week 0) and during immunotherapy. Binding of CD4+ T-cells to the control hCLIP-HLA-DRB1*1501 tetramer is shown in the lower line of dot-plots. Numbers in the top right corner of dot-plots is tetramer binding cells as a percentage of CD4+ T-cells. B. Antigen specific T-cell frequencies were followed in 6 HLA-DRB1*1501-positive individuals using HLA-DRB1*1501 Ves v 5 peptide-tetrameric complexes. Frequencies were expressed as a percentage of CD4+ T cells. There was a significant reduction (p<0.05) in the frequency of tetramer positive T-cells from weeks 4 to 7 using Student's paired t-test. C. Tetramer binding to the control hCLIP-HLA-DRB1*1501 tetramer was also followed in 4 of the individuals during immunotherapy. There was no significant change in the frequency of hCLIP-tetramer binding cells, p = 0.53, one-way ANOVA.

**Figure 6:**
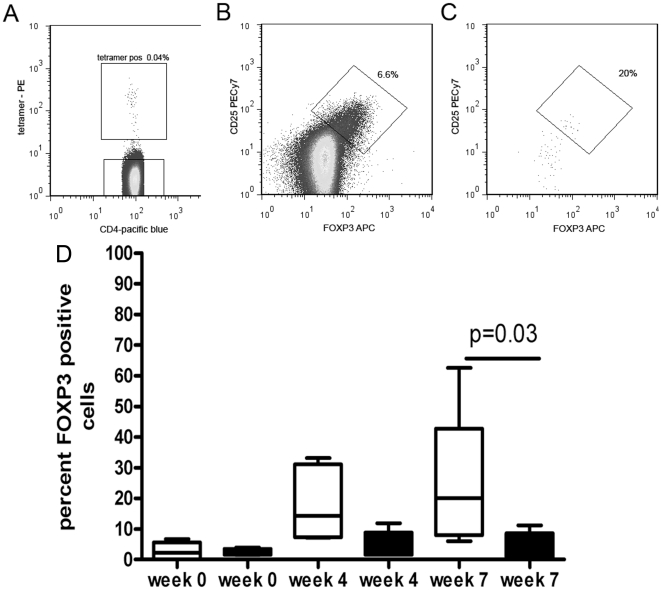
Longitudinal change in FOXP3+ T-cells during immunotherapy. A. Example of dot-plot showing frequency of HLA-DRB1*1501 Ves V 5 peptide-tetramer binding cells from wasp allergic patient 7 weeks after immunotherapy. FOXP3+ CD25hi T-regs population were visualized in the tetramer negative population and were gated as shown in B. This T-reg gate was used to identify antigen-specific tetramer-binding T-regs as shown in C. D. The percentage of FOXP3 expressing cells within the tetramer-binding subset (open boxes) and the total CD4+ population (filled boxes) were determined during immunotherapy in 6 DRB1*1501 wasp allergic patients.

These data showed that IL-4 producing wasp venom-specific T cells are initially induced during immunotherapy, but after weeks 3-5 the functional IL-4 responses are progressively lost through to week 12. However, it is not clear whether this represents loss of circulating specific cells and/or change in cell function. In order to address this question, we used the two Ves v 5/DRB1*1501 tetrameric complexes to track epitope specific T cells over time in 6 of the wasp venom allergic patients who expressed HLA-DRB1*1501. Frozen PBMC taken from an individual at different stages of immunotherapy were thawed and stained with tetramer at the same time, thus limiting any time related degradation of tetramer or other variation related to analyses taken at different times. We would have liked to have investigated the longitudinal frequency of tetramer binding T-cells in HLA-DRB1*1501 wasp allergic individuals not receiving immunotherapy but as our cohort all undergo immunotherapy we instead demonstrated that the frequency of tetramer binding CD4 T-cells did not significantly change with time in healthy individuals not receiving immunotherapy (Figure S5). HLA peptide tetrameric complex binding to T cells is not dependent on particular functional activity and will therefore detect all epitope-specific T cells regardless of functional subset. Figure 5A gives an example of Ves v 5 HLA-DRB1*1501 tetramer binding of CD4 T-cells in a wasp allergic individual during immunotherapy alongside the frequency of CD4+ T-cells binding to a control hCLIP-HLA DRB1*1501 tetramer in the same individual. In figures 5B and C *ex vivo* Ves v 5 tetramer and control hCLIP tetramer binding was followed in 6 individuals longitudinally and confirms a similar initial induction to weeks 4, followed by a loss of cells to a level not significantly different to the start of immunotherapy. The frequency of CD4+ T-cells binding to a control hCLIP-HLA DRB1*1501 tetramer in the same individuals did not change significantly (Figure 5B). Whilst this implicates a relative loss of specific T cells during later immunotherapy, it does not indicate phenotypic or functional characteristics of the remaining cells. Figure 6 (A and B) shows identification of antigen-specific tetramer-binding T-regs by gating on FOXP3-positive and CD25hi as shown in the tetramer negative population. Figure 6D shows that the proportion of Ves v 5-specific T cells that were FOXP3-positive also increased over time compared to the tetramer-negative population and this proportion was maintained to week 8 of immunotherapy. Furthermore, we observed that the proportion of tetramer-positive cells that expressed CD45RO was significantly lower at week 4 and week 8 compared to pre-treatment levels (data not shown).

**Figure 7:**
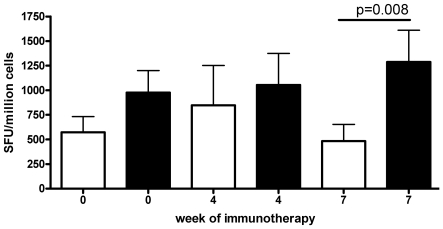
IL-10 is inhibiting IL-4 responses in Ves v 5 specific lines after immunotherapy. Production of IL-4 by day 14 cultured T cells in response to recombinant Ves v 5 at different stages of immunotherapy in the absence (open boxes) or presence (filled boxes) of anti-IL-10. Addition of anti-IL-10 at week 7 restored the frequency of rVes V 5 specific IL-4 T-cells (p = 0.008, Student's paired t-test).

FOXP3 is expressed at constitutively high levels on natural T regulatory cells but can be induced on both effector T cells and on acquired T regulatory cells[62]–[63]. However little is known of the relationship between the latter two populations and whether they originate from common precursor cells. The frequency of Ves v 5-specific T cells was too low *ex vivo* for use in suppression assays, but we were able to investigate whether the fall in antigen-specific T cells was IL-10 dependent. We observed that the addition of anti-IL-10 restored the proliferation and IL-4 production of Ves v 5-specific T cell during the course of immunotherapy (Figure 7). Indeed, we observed significant (P<0.005) further enhancement when anti-CD25 was used to deplete PBMC of CD25hi T-regs in addition to the anti-IL-10 (Figure S6). Taken together with the FOXP3 findings, these data argue in favour of induction of acquired antigen-specific FOXP3-positive regulatory T cells and IL-10 secreting regulatory T cells during wasp venom immunotherapy which influence the frequency and function of venom-specific IL-4 producing effector T cells. Indeed, allergen specific IL-10 secreting cells have been demonstrated to occur in healthy controls and in bee-keepers.

**Figure 8:**
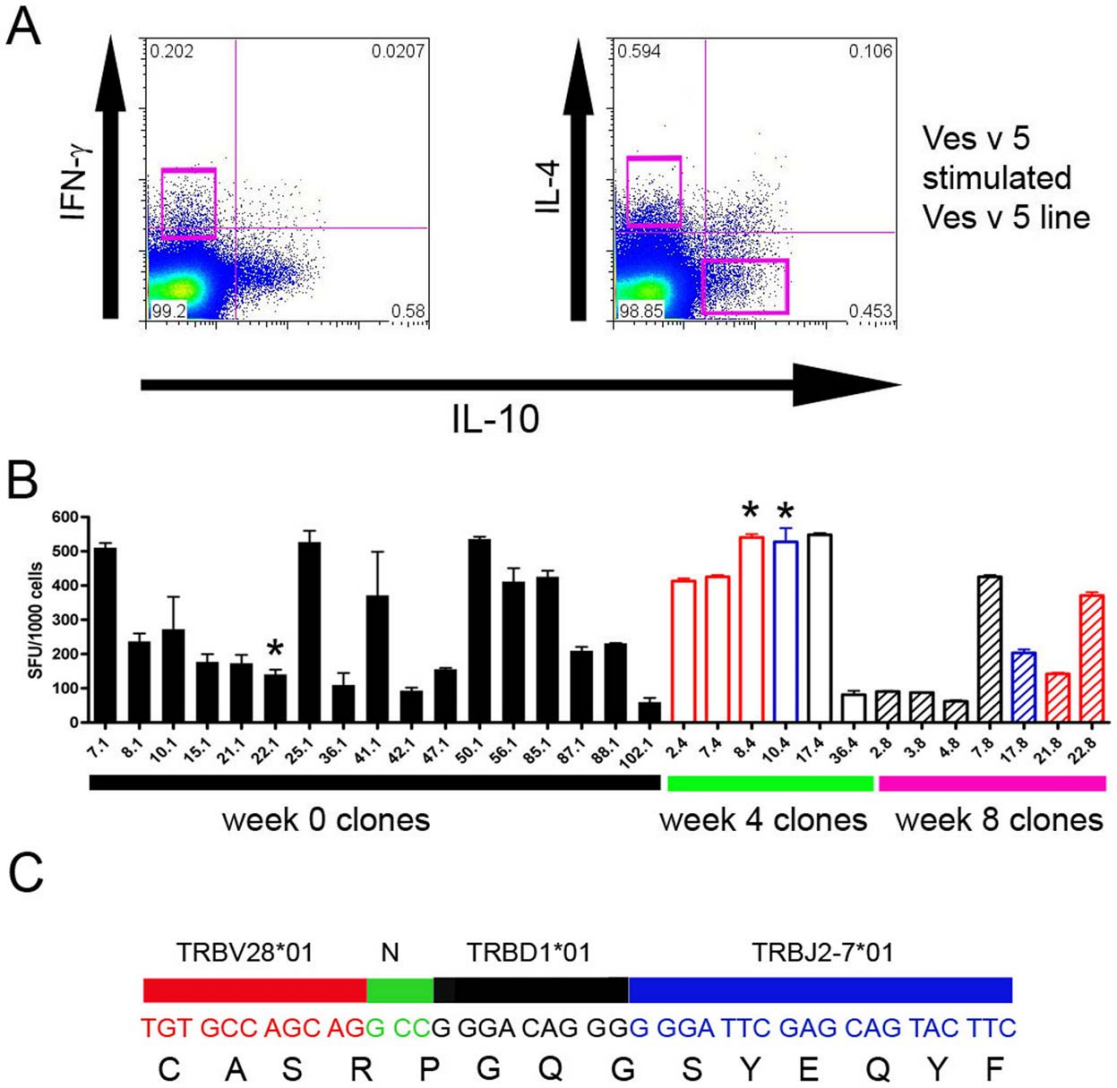
Identification and TCR sequencing of Ves v 5 specific T-cell clones during immunotherapy. A. IFN-γ, IL-4 and IL-10 secreting cells were sorted using a cytokine secretion assay from day 14 cultured Ves v 5-specific T cell populations. Clones were established by in vitro expansion of single cytokine secreting cells. B. Cytokine secretion in response to antigen 5 stimulation was measured by ELISpot using IL-4 (black boxes), IFN-γ (red boxes) and IL-10 (blue boxes) derived clones, sorted before immunotherapy (week 0) and at weeks 4 and 8 of immunotherapy. Sfu spot forming units. C. Clones 22.1, 8.4 and 10.4 (*) shared an identical TCR Vbeta sequence.

However the data do not address the origin of the acquired regulatory T cells and whether they arise from the IL-4 producing effector T cells or whether they represent a distinct lineage that is induced during immunotherapy. In order to address this question, we used cytokine capture assays to isolate Ves v 5-specific T cells on the basis of IL-4, IFNγ and IL-10 production over time. The T cell receptor genes of the clones were then sequenced in order to determine the nature of lineage consistencies. We used a whole Ves v 5 stimulation approach in order to avoid bias towards one particular epitope. Many clones were identified (Figure 8), but surprisingly we observed one clone that emerged from one individual at 2 separate time points derived from different populations of cytokine production. At week 1, the clone had been isolated on the basis of IL-4 production in response to the entire overlapping Ves v 5 peptide pools. However by week 4, the clone was not isolated from the IL-4 producing population, but was instead isolated from both the IL-10 and IFNγ producing subsets. Furthermore, we went on to observe that the clone recognised our peptide NDFLKTGHYTQMVWA and stained with the relevant tetramer. These data show that a single TCR precursor can give rise to IL-4, IFNγ or IL-10-producing cells at different stages of immunotherapy that accompanies acquisition of clinical tolerance. Taken together with the tetramer and FOXP3 data documented above, these findings would be compatible with antigen-specific acquired regulatory T cells being derived from a common precursor to effector T cells in humans.

## Discussion

Wasp venom immunotherapy presents an excellent opportunity to understand the acquisition of clinical tolerance in humans. The administration of weekly subcutaneous injections of wasp venom to humans is associated with changes in systemic immune responses with consequent well-documented clinical efficacy. Investigation of the underlying mechanisms carries significant implications for understanding peripheral tolerance as well as leading to potential future therapeutic developments in the fields of allergy and other diseases such as autoimmunity and transplantation.

A key step in the investigation of T-cell responses is to identify T-cell antigens, map epitopes and determine HLA-restriction. Having previously identified the major T-cell antigenic sources in wasp venom as Ves v 1, Ves v 2 and Ves v 5 [64], we have now identified 2 HLA DRB1*1501 restricted epitopes within Ves v 5 using T-cell lines from wasp allergic individuals. Bohle et al also identified T-cell epitopes in 13 wasp allergic patients and 13 healthy controls by using overlapping synthetic peptides, although they did not did not determine HLA restriction [53]. Interestingly, T-cell clones from three of their HLA-DRB1*1501 expressing allergic patients recognised peptides containing the 2 HLA-DRB1*1501 T-cell epitopes that we identified.

The identification and fine mapping of Ves v 5 epitopes also allowed for the development of HLA-DRB1*1501-Ves v 5 peptide tetrameric complexes, and through their subsequent use we were able to identify antigen specific T-cells in both allergic individuals and healthy controls. Although the frequency of tetramer positive cells was very low ex vivo (0.01–0.09% of CD4+ T cells), it is comparable to frequencies of tetramer positive cells detected in cat allergy and after varicella zoster exposure [65]-[66]. The wasp allergic patients had higher frequencies of allergen specific T-cells as defined by tetramer binding and functional cytokine responses, which has been similarly observed in cat and pollen allergy [66]–[67]. We hope that the identification of wasp venom specific epitopes and the consequent availability of wasp venom-specific HLA tetrameric complexes will aid such future monitoring in the further development of wasp venom immunotherapy, with lessons for other diseases.

By mapping constituent epitopes within a dominant antigen of wasp venom, we have been able to take a new approach to the characterization of T cell correlates of successful immunotherapy in wasp venom allergic individuals. In contrast to previous studies, we have shown that during the first 3–5 weeks of immunotherapy there is an induction of effector T cells, which is then later followed by a relative loss of such cells in the group of 13 wasp-venom patients from whom we longitudinally tracked cytokine responses. We have also observed that immunotherapy is associated with the acquisition of IL-10-producing antigen-specific cells that can derive from a common precursor to effector T cells. Lastly the fall in antigen-specific T cells during late immunotherapy is partially IL-10 dependent.

The early induction of IL-4-producing antigen-specific T cells in the first few weeks of immunotherapy would fit with observations of late asthmatic reactions in individuals receiving peptide immunotherapy for cat allergen reactive asthma [45]–[50]. It might also contribute to reactions observed in other immunotherapeutic uses of peptide [68]–[69]. It has previously been well argued that such reactions may be dose dependent and indeed there are likely to be therapeutic strategies that reduce the associated risk [51]. Clearly an understanding of such an early induction of effector T cells will be crucial for the progression of peptide immunotherapy approaches. It is perhaps unsurprising that administration of an antigen leads to induction of immune responses, and indeed this is the aim of current vaccination approaches. However, the causes of the subsequent reduction in allergen specific reactivity despite antigen dose escalation, is key to understanding mechanisms of clinical tolerance.

We observed that the later fall in IL-4 producing wasp venom-specific T cells was associated with a loss of tetramer-binding cells in the 6 HLA-DRB1*1501 wasp allergic patients from whom we had longitudinal samples. This argues that there is not simply a change of function of antigen-specific T cells, but also a reduction in the circulating frequency of reactive T cells. Although we cannot exclude the redistribution of antigen-specific T-cells from the periphery, it is also possible that activation induced cell death is accounting for this loss. One limitation of the tetramer approach is that for CD4+ T cells, it is necessary to gate out dying cells, which means that investigation of mechanisms of cell death in the tetramer-binding population is problematic. Sorting of tetramer-binding cells also potentially introduces artefacts which have limited our ability to address this question. Nevertheless it is clear that there is a fall in wasp venom-specific T cells between weeks 4 and 8 of immunotherapy which associates with a reduction in antigen-specific IL-4 production.

The fall in IL-4 production by wasp venom specific T cells during late immunotherapy is accompanied by a rise in the proportion of remaining allergen-specific T cells that produce IL-10. Furthermore the proportion of the tetramer-binding cells that express FOXP3 also increases, and the loss of IL-4 production is partially IL-10 and CD4+CD25+ cell dependent. These data provide evidence supporting the possibility that IL-10-producing wasp venom-specific FOXP3-positive T cells are induced during venom immunotherapy and influence antigen-specific production of the effector Th2 cytokine IL-4. The lineage specificity of IL-10-producing acquired T regulatory cells has been debated, but we show that similar cells develop during immunotherapy and can share a common precursor with pre-treatment effector Th2 cells in humans. This suggests that repeated subcutaneous administration of antigen in the absence of concurrent inflammation can lead to either the switch of existing effector IL-4 producing T cells to IL-10 production or to the preferential outgrowth of IL-10 producing cells with a shared common precursor. Akdis et al have also recently shown that in bee-keepers who are exposed to high doses of bee venom, there is evidence of clonality accompanying the switch to IL-10 secreting T-cells [70].

It is possible that the observed venom-specific IL-10 producing cells and FOXP3-positive cells represent an epiphenomenon and are not relevant to induction of tolerance, but the accumulating data on regulatory T cells and their relevance to many diseases, suggest that these are likely to prove to be important cells in the success of immunotherapy [71]–[73]. That IL-10 may play a role in allergen tolerance has been suggested by a previous study that demonstrated increased frequencies of allergen specific IL-10 secreting T-cells in healthy controls compared to allergic individuals by ELISpot [74]. It will be of interest to examine the frequencies of such cells in the subgroup of individuals who lose clinical tolerance after many years of immunotherapy. Furthermore, the data do not address how the IL-10-producing FOXP3-positive cells might influence the IgE-dependent clinical reactivity, but the known IL-10 induced class switching to IgG4 in humans may be relevant. Indeed, among laboratory animal handlers and children, reduced prevalence of laboratory animal and cat allergy, respectively, is associated with high serum concentrations of allergen specific IgG4 and high animal exposure [75]–[76]. Interestingly, we also observed a significant rise in wasp- venom-specific IgG4 after 8 weeks of immunotherapy consistent with previous reports [4]–[6]. Further longitudinal studies on the durability of Ves v 5 - specific T cells may help determine the optimal duration of immunotherapy, which is empirically determined at 3 to 5 years at present.

In summary, we have developed tools to facilitate the tracking of T cells during clinical tolerance induction in humans, and use these to show that wasp venom immunotherapy associates with an initial induction of IL-4-producing wasp venom-specific T cells, which is followed after 3–5 weeks by a fall in the frequency of cells and a shift towards greater IL-10 production and FOXP3 expression. Furthermore, the IL-10 producing cells can share a common precursor with pre-treatment IL-4-producing T cells suggesting that acquired regulatory T cells in humans can be derived from similar populations to effector T cells. The data contribute to our understanding of clinical tolerance acquisition in humans with consequent potential therapeutic implications in the setting of allergy and other disease. We hope that the development of such tools will be of value to those investigating allergen-specific immune responses and mechanisms of tolerance induction.

## Materials and Methods

### Ethics Statement

The study was approved by Oxfordshire Research Ethics Committee (REC) B - reference C02.291. All participants gave written informed consent.

### Subjects

Twenty three individuals with a clinical history of wasp venom systemic allergic reactions and positive wasp venom specific skin tests and/or circulating IgE detected by RAST who were to begin an 8-weeks modified rush protocol of Vespula sp. Venom (ALK-Abello) immunotherapy were recruited through the venom clinic at the Churchill Hospital. Wasp allergic subjects were bled before (week 0) and after (weeks 1–12) immunotherapy. 14 healthy volunteers with a history of previous wasp stings, but without a history of wasp venom allergy were also used. Details of the dose of wasp venom that was administered subcutaneously were at weekly intervals as follows: 3 µg, 5 µg, 10 µg, 20 µg, 40 µg, 65 µg, 100 µg, 100 µg. Peripheral blood mononuclear cells (PBMCs) were separated from heparinized peripheral blood by density gradient using Lymphoprep (Nycomed, Roskilde, Denmark). PBMCs were then washed in RPMI supplemented with penicillin, streptomycin and l-glutamine (R0) and resuspended in RPMI with penicillin, streptomycin, l-glutamine and 10% fetal calf serum (FCS; R10).

#### HLA Typing

All patients and laboratory volunteers were HLA typed. Genomic DNA Puregene DNA isolation kit (Gentra Systems, USA) was used to isolate DNA from whole blood. HLA-A, -B, -C, DRB1, DRB3, DRB4, DRB5 and DQB1 specificities were determined using sequence specific primers by our in-house HLA typing service.

### Antigens

Ex-vivo ELISpot assays used Vespula species wasp venom (Vespa Labs, Spring Mills, PA, USA) at 5 µg/ml and also synthetic peptide pools. 20 mer peptides overlapping by 10 amino acids which spanned the whole length of the Ves v 5 protein were synthesized in house in an automated synthesizer using 9-fluorenylmethoxycarbonyl chemistry. The purity of the peptides was determined to be greater than 90% by high-pressure liquid chromatography analysis and mass spectrometry. Individual Ves v 5 peptides were pooled to create 4 peptide pools with each pool containing 5 peptides (peptide concentration of 20 µM). Recombinant Ves v 5 was a kind gift from R Suck (Allergopharma, Germany) and was used to expand T-cell lines in vitro at 1 µg/ml and for stimulation of T-cell lines at 100 µg/ml.

### ELISpot

ELISpot plates (Millipore Corp., Bedford, MA, USA) were coated with either anti-human interferon (IFN)-γ, IL-4 or IL-10 antibody overnight (Mabtech AB, Nacka, Sweden). The plates were washed six times with RPMI-1640 and blocked for 1 h with RPMI-1640 supplemented with 2 mM L-glutamine, 100 IU/ml penicillin and 100 µg/ml plus 10% human serum (R10*). For *ex-vivo* responses, 0.2×10^6^ PBMC were stimulated with Vespula species venom (final concentration 5 µg/ml) in 96 well ELISpot plates. For ELISpot reponses using T-cell lines, 40,000 cells were added to each well to which Ves v 5 peptide pools or individual peptides were added (final concentration 20 µM). Wells were set-up in duplicate. Phytohaemagglutinin was included as a positive control and R10* alone was included as a negative control. After overnight incubation at 37°C and 5% CO2, plates were washed x6 in PBS-Tween 0.05% and incubated with 1 µg/ml of biotin-linked anti-IFN-γ, IL-10 or IL-4 monoclonal antibody (mAb) (Mabtech AB) for 2 hours. After washing x 6 in PBS-Tween 0.05%, the plates were incubated for a further 1 hour with streptavidin-alkaline phosphatase (Mabtech AB). Spots were visualized using an alkaline phosphatase conjugate substrate kit (Biorad, Hercules, CA, USA) and enumerated using an automated ELISpot reader. Results were expressed as spot-forming cells per total number of PBMCs after subtracting the background (cells alone).

### T-cell lines

Antigen 5 specific T-cells were expanded in vitro by incubating 4×10^6^ PBMCs with recombinant Ves v 5 (10 µg/ml) in 2 mls R10* in 24 –well plates for 10 days (Corning, ). Interleukin-2 was added on days 3 and 7 at a concentration of 100 units/ml. Anti human IL-10 (Peprotech Inc, Rocky Hill, NJ, USA) was also added to certain lines on day 0, 3 and 10 to maintain a concentration of 4 µg/ml. All cell lines were maintained at 37°C, in 5% CO2. T cell lines were washed twice in PBS 24 hours before their use in ELISpot and cytokine secretion assays.

### Cytokine secretion assay

5×10^6^ recombinant Ves v 5 T-cell lines were stimulated with 10 µg/ml recombinant Ves v 5 in 1 ml of R10* in 24 well plates for 6 hours at 37C in humidified 5%CO2. Cells were then harvested and labelled with anti–IFNγ/CD45, anti–IL-4/CD45, and anti–IL-10/CD45 Ab–Ab conjugates according to the manufacturer's protocol (Miltenyi Biotec, Germany) for 5 min at a concentration of 10^7^ cells/ml in ice-cold phosphate buffered saline pH 7.2, containing 0.5% bovine serum albumin (BSA) and 2 mM EDTA (PBS-EDTA). The cells were diluted with 37°C R10* medium to a final concentration of 10^6^ cells/ml and were slowly rotated at 37°C for 45 minutes. The cells were then washed in PBS-EDTA at 4°C and then stained with FITC-conjugated anti–IFN-γ (Miltenyi Biotec) APC-conjugated anti–IL-10 (Miltenyi Biotec), PE-conjugated anti–IL-4 (Miltenyi Biotec), Pacific Blue conjugated anti-CD4 (BioLegend, CA, USA) and vital dye Via-probe (BD PharMingen) for 10 min at 4°C. After washing, cells were re-suspended in PBS 0.5% formaldehyde and acquired on a CyAn™ (DakoCytomation, Glostrup, Denmark) flow cytometer. For cell sorting cells were re-suspended in PBS and sorted on a MoFlo™ (Beckman Coulter, Fullerton, CA,USA).

### Tetramer staining

DRB1*1501 iTAg MHCII tetramer was purchased from Beckman Coulter (Hialeah, FL, USA). DRB1*1501-PE tetramer was complexed to Ves v 5 peptides NDFLKTGHYTQMVWA and HKHYLVCNYGPSGNFKNEEL. hCLIP peptide HLA DRB1*1501 negative control tetramer was provided by the NIH Tetramer Core Facility (Emory Vaccine Center, Atlanta, GA). Cell lines and PBMC were incubated with 2 µg/ml HLA class II tetramer for 60 min at 37°C in R10* before staining with cell surface marker antibodies at room temperature for 20 minutes, including CD4-pacific blue (Biolegend, San Diego, CA, USA), CD14-PerCP, CD45RO APC, CD19-PerCP and vital dye ViaProbe (all BD Pharmingen, Oxford, UK) were added for 20 min at room temperature. Stained cells were washed with phosphate-buffered saline (PBS) and fixed in 0•5% PBS/formaldehyde. For FOXP3 staining tetramer and cell surface marker stained cells were then fixed permeabilised and stained with anti-FOXP3 according to the manafacturer's instructions (eBiosciences, clone PCH101). Cells were acquired on a CyAn™ (DakoCytomation) and analysed using FlowJo software. Dead and CD14 and CD19 positive cells were excluded from the analysis.

### CD25 depletion

CD4+ CD25+ regulatory T-cells were depleted using anti-CD25hi magnetic bead conjugated antibodies (Invitrogen Dynal AS, Oslo, Norway) from PBMC from patients before and 12 weeks after immunotherapy. Conjugated beads were washed x3 in PBS-0.1% BSA using a magnet to remove the supernatant. Beads (400 million beads/ml) were then mixed with PBMC (25 million/ml) and incubated at 4C for 30 min with gentle rolling. Labelled cells were then removed using a magnet. Depletion of CD4+ CD25+ T-cells was confirmed by flow cytometry (data not shown).

### T-cell cloning

IL-4. IL-10 and IFN-γ secreting Ves v 5 specific CD4+ T-cells were identified using the cytokine secretion assay and single cells were sorted into individual well in round-bottomed 96-well plates using a MoFlo cell sorter. Tetramer staining cells of PBMC stimulated for 10-days with relevant peptide (2 µM) were also similarly sorted by flow cytometry. 100 µl of irradiated feeder cells – (1∶1∶1 of PBMC from 3 different individuals) at 1×10^6^ cells/ml in R10* containing IL-2 (100 IU/ml) and PHA (1 mg/ml) were added to each well. Clones were fed every 3–4 days with R10* containing IL-2 (100 IU/ml) and were re-stimulated with feeder cells every 2–3 weeks.

### TCR Sequencing

RNA was extracted from CD4 clones using TRIzol LS reagent (Invitrogen,Carlsbad, California, USA). Full length cDNA was generated using a switching mechanism At 5′ end of RNA Transcript (SMART) (Clontech) using Superscript 3 reverse transcriptase (Invitrogen), oligo(dT) (Invitrogen), and SMART II™ A oligonucleotide (5′-AAGCAGTGGTATCAACGCAGAGTGrGrGr-3′, where Gr is a ribonucleotide) (Clontech, Mountain View, CA, USA). The cDNA was subsequently used as a template for 5′ rapid amplification of C-terminal ends (5′-RACE) using 5′ universal primers (Clontech), anti-sense TCR beta chain constant region primer, TCRB 5′-ATTCACCCACCAGCTCAGCTCCACG-3′ and a high-fidelity polymerase Advantage 2 (Clontech) and incorporating a touch-down polymerase chain reaction (PCR) protocol. CDR3 regions were sequenced using the TCRB primer. TCR V, D and J regions were identified using the International Immunogenetics Information system (IMGT) database [77]–[78].

### Statistics

Appropriate statistical tests were used to determine if the null hypothesis could be rejected at a probability of <0.05. Paired t-test, one-way ANOVA and Chi square test were used to establish significance of differences between data-sets. All analysis was performed using the statistical software package GraphPad Prism 4.

## Supporting Information

Figure S1 *Ex vivo* IL-4 ELISpot responses to Ves v 5 peptide pools in 23 wasp allergic patients: in A, 92 responses representing summed responses to 4 Ves v 5 pools were determined ex-vivo. The horizontal dashed bar represents the cut-off for a positive response (mean + 3x standard deviation of negative control wells). Mean response is 12.4 SFU (spot forming units) per million PBMC (solid line); whereas in B responses to each peptide pool are shown, the horizontal bars representing the means for each peptide pool and the cut off for a positive response is the horizontal dashed line.
(0.45 MB TIF)

Figure S2 IL-4 and IFN-γ peptide responses after 10 day in vitro expansion with recombinant Ves v 5 (1 µg/ml) followed by overnight stimulation with Ves v 5 peptide pools at final concentration of 10 µm. Responses within boxes were above the cut-off (dashed line), which was determined as mean of negative control plus 3x standard deviation: 249 for IL-4 and 204 for IFN-γ. The mean frequency of Ves v 5 peptide specific IL-4 secreting T-cells was higher in wasp allergic patients than in controls: (mean 1032±129.9 SFU per million cells N = 23 for wasp allergics and 353.1 SFU per million cells ±54.34 N = 8 for controls (p = 0.029). There was no significant difference between the frequency of IFN-γ positive responses between venom allergics and controls, 781.3±145.0 N = 16 wasp allergics, 481.9±95.13 N = 8 controls p = 0.18.
(4.52 MB TIF)

Figure S3 Longitudinal IFN- γ response to control antigen in 4 wasp allergic individuals receiving immunotherapy. Ex-vivo responses to FEC peptide pool was determined by ELISpot in wasp allergic individuals during immunotherapy. There was no significant difference between the mean responses. 1-way ANOVA p = 0.93.
(0.81 MB TIF)

Figure S4 Longitudinal venom-specific IgG4 responses during immunotherapy in 6 HLA-DRB1*1501 individuals before (week 0) and after immunotherapy (weeks 4 and 7). There was a significant increase in serum IgG4 concentration after 4 and 7 weeks of immunotherapy (Student's paired t-test).
(0.21 MB TIF)

Figure S5 Frequency of tetramer specific T-cells was enumerated by flow cytometry in 4 HLA DRB1*1501-positive non-wasp allergic individuals who did not undergo immunotherapy at time point A and timepoint B (4–6 weeks later). There was no significant difference in the frequency of tetramer binding T-cells, p = 0.69, student's paired t-test.
(0.25 MB TIF)

Figure S6 (A) Short term T-cell lines to rVes v 5 were generated in 4 wasp allergic individuals at week 12 of immunotherapy in the presence of anti-IL-10 (diagonal stripes) and after magnetic bead depletion of CD25hi T-regs(filled). T-cell lines were then stimulated overnight with Ves v 5 peptide pools (10 µM) and IL-4 responses were detected by ELISpot. The results are expressed as means and SD spots per million cells. (B) Short term lines were also generated to FEC peptide pool with anti-IL10 and CD25 depletion and re-stimulated overnight with FEC peptide pools on IFN-γ ELISpot plates. Mean and SD spot forming units per million cells are shown.
(0.88 MB TIF)
