# Supplementary figures and images for: Tracking Antigen-Specific T-Cells during Clinical Tolerance Induction in Humans
Source: PLoS One. 2010 Jun 9;5(6):e11028. doi: 10.1371/journal.pone.0011028 (PMC2882953; doi:10.1371/journal.pone.0011028)

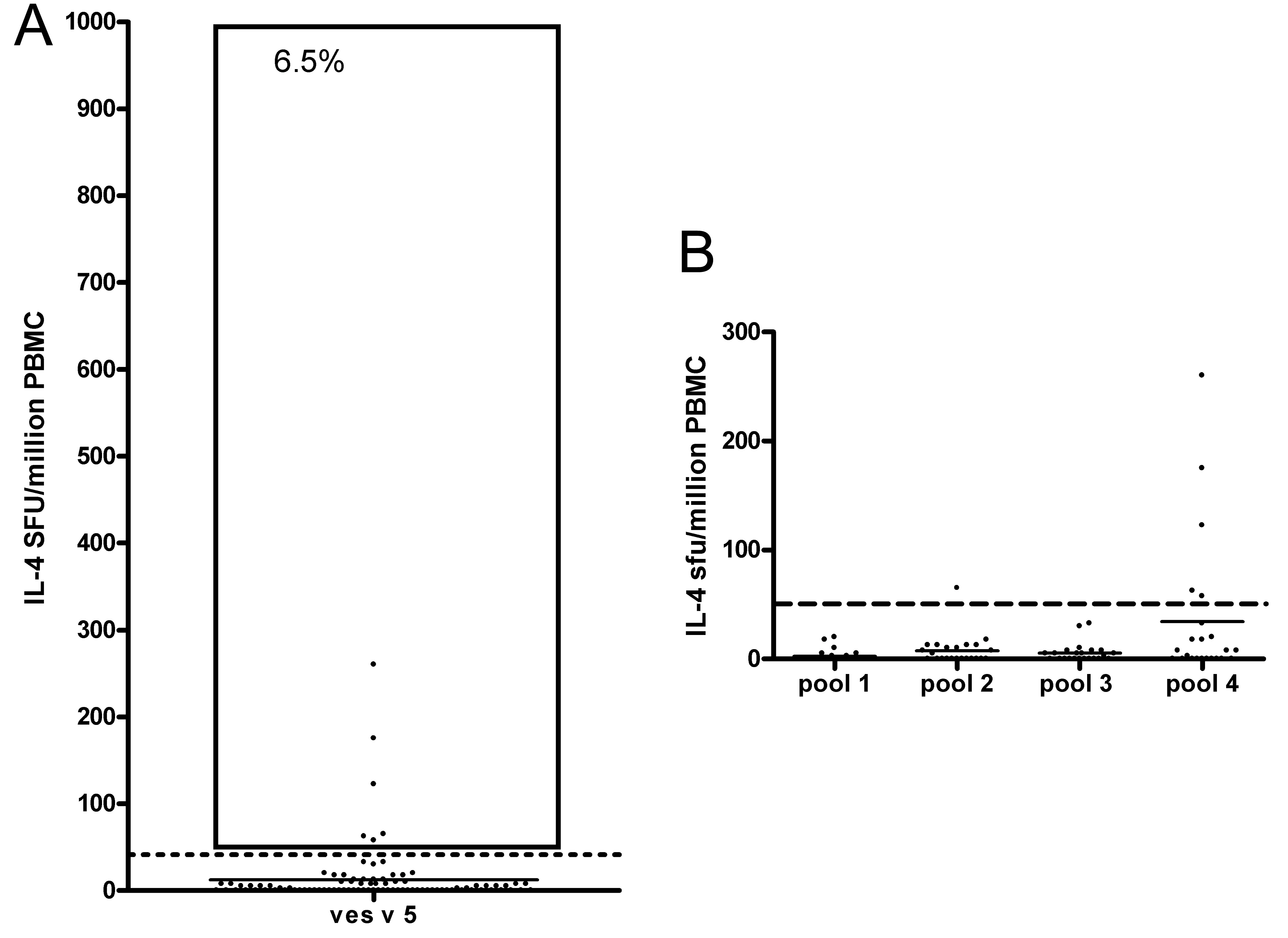

Supplement: Figure S1 — Ex vivo IL-4 ELISpot responses to Ves v 5 peptide pools in 23 wasp allergic patients: in A, 92 responses representing summed responses to 4 Ves v 5 pools were determined ex-vivo. The horizontal dashed bar represents the cut-off for a positive response (mean + 3x standard deviation of negative control wells). Mean response is 12.4 SFU (spot forming units) per million PBMC (solid line); whereas in B responses to each peptide pool are shown, the horizontal bars representing the means for each peptide pool and the cut off for a positive response is the horizontal dashed line. (0.45 MB TIF) [file pone.0011028.s001.tif]

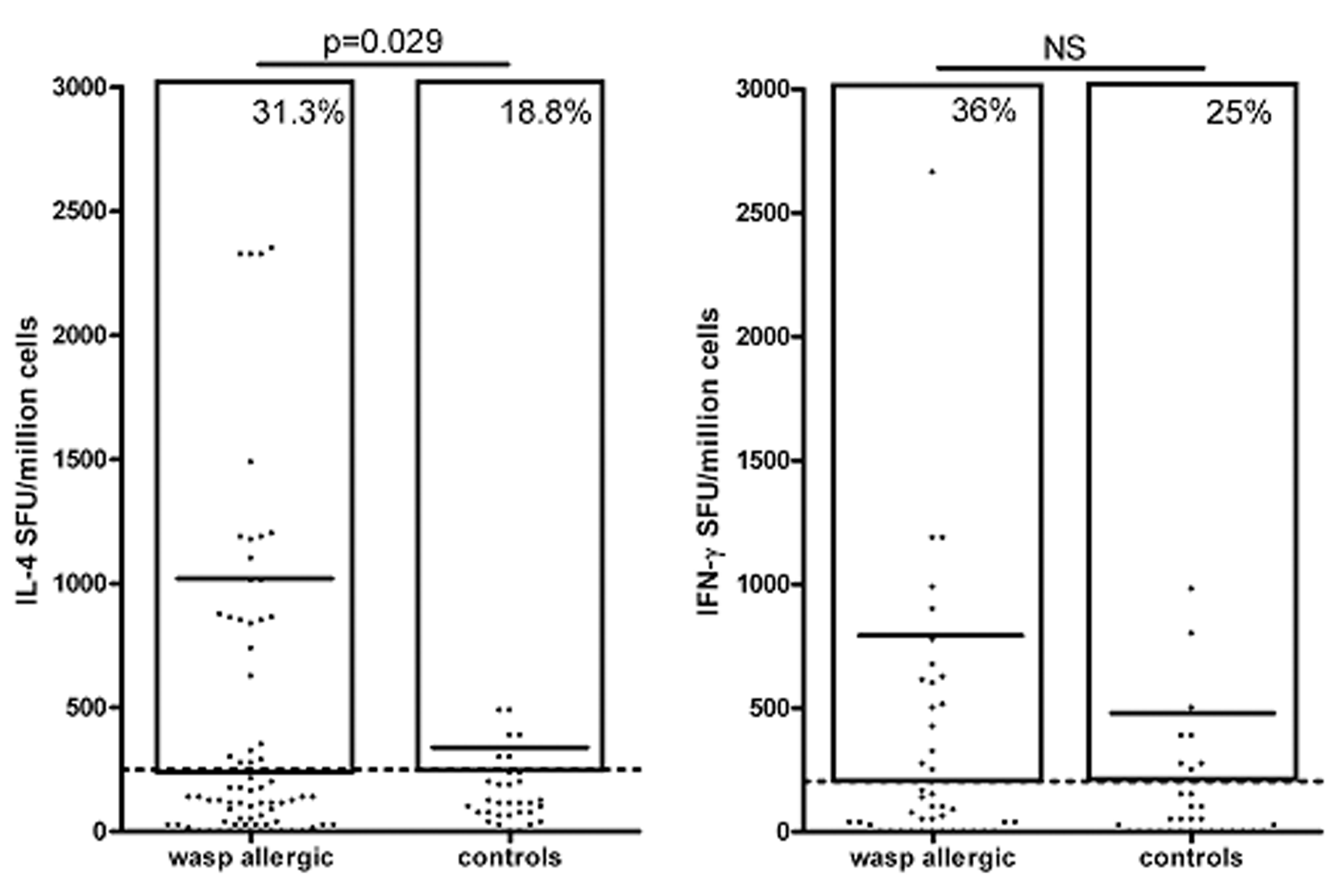

Supplement: Figure S2 — IL-4 and IFN-γ peptide responses after 10 day in vitro expansion with recombinant Ves v 5 (1 µg/ml) followed by overnight stimulation with Ves v 5 peptide pools at final concentration of 10 µm. Responses within boxes were above the cut-off (dashed line), which was determined as mean of negative control plus 3x standard deviation: 249 for IL-4 and 204 for IFN-γ. The mean frequency of Ves v 5 peptide specific IL-4 secreting T-cells was higher in wasp allergic patients than in controls: (mean 1032±129.9 SFU per million cells N = 23 for wasp allergics and 353.1 SFU per million cells ±54.34 N = 8 for controls (p = 0.029). There was no significant difference between the frequency of IFN-γ positive responses between venom allergics and controls, 781.3±145.0 N = 16 wasp allergics, 481.9±95.13 N = 8 controls p = 0.18. (4.52 MB TIF) [file pone.0011028.s002.tif]

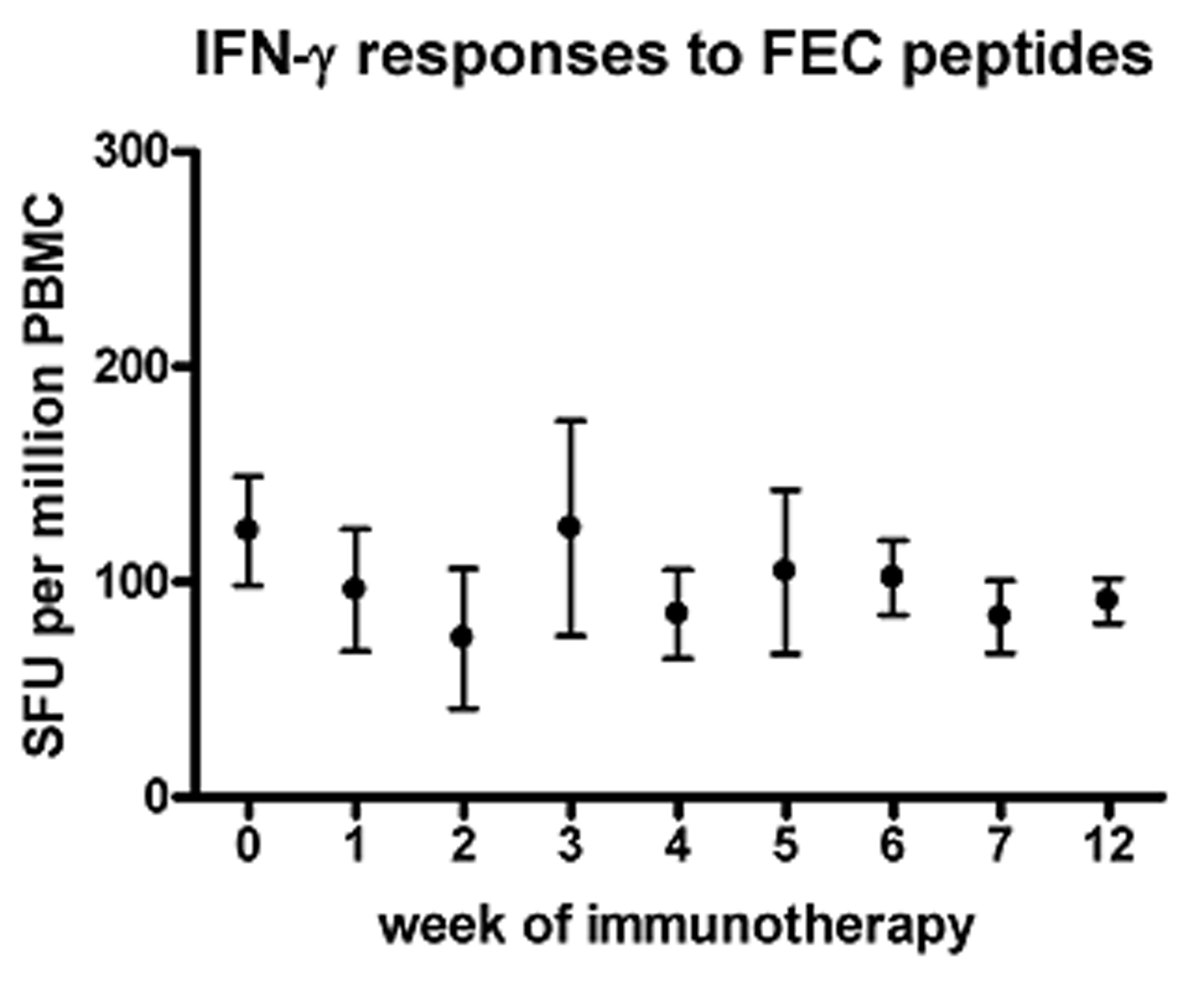

Supplement: Figure S3 — Longitudinal IFN- γ response to control antigen in 4 wasp allergic individuals receiving immunotherapy. Ex-vivo responses to FEC peptide pool was determined by ELISpot in wasp allergic individuals during immunotherapy. There was no significant difference between the mean responses. 1-way ANOVA p = 0.93. (0.81 MB TIF) [file pone.0011028.s003.tif]

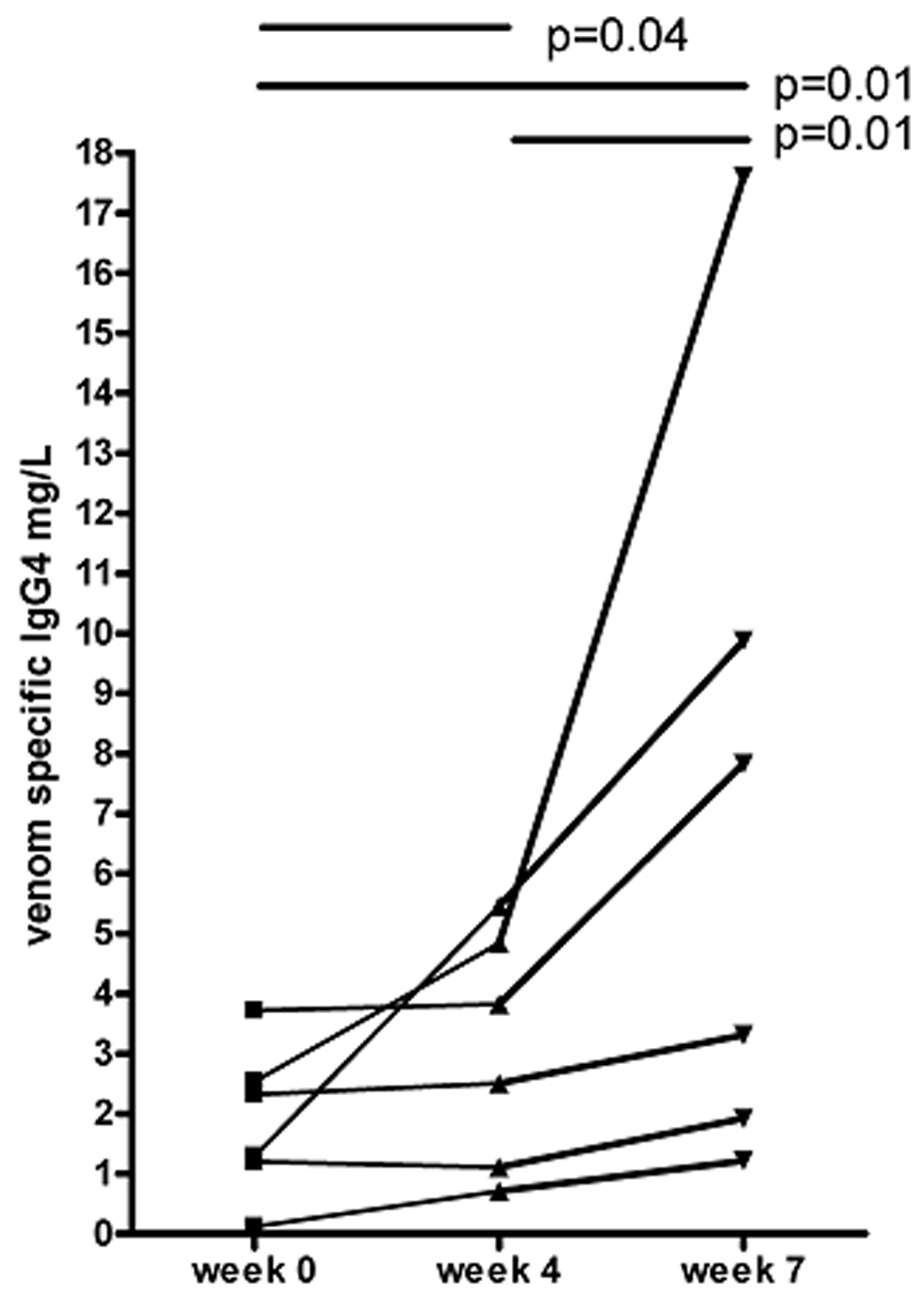

Supplement: Figure S4 — Longitudinal venom-specific IgG4 responses during immunotherapy in 6 HLA-DRB1*1501 individuals before (week 0) and after immunotherapy (weeks 4 and 7). There was a significant increase in serum IgG4 concentration after 4 and 7 weeks of immunotherapy (Student's paired t-test). (0.21 MB TIF) [file pone.0011028.s004.tif]

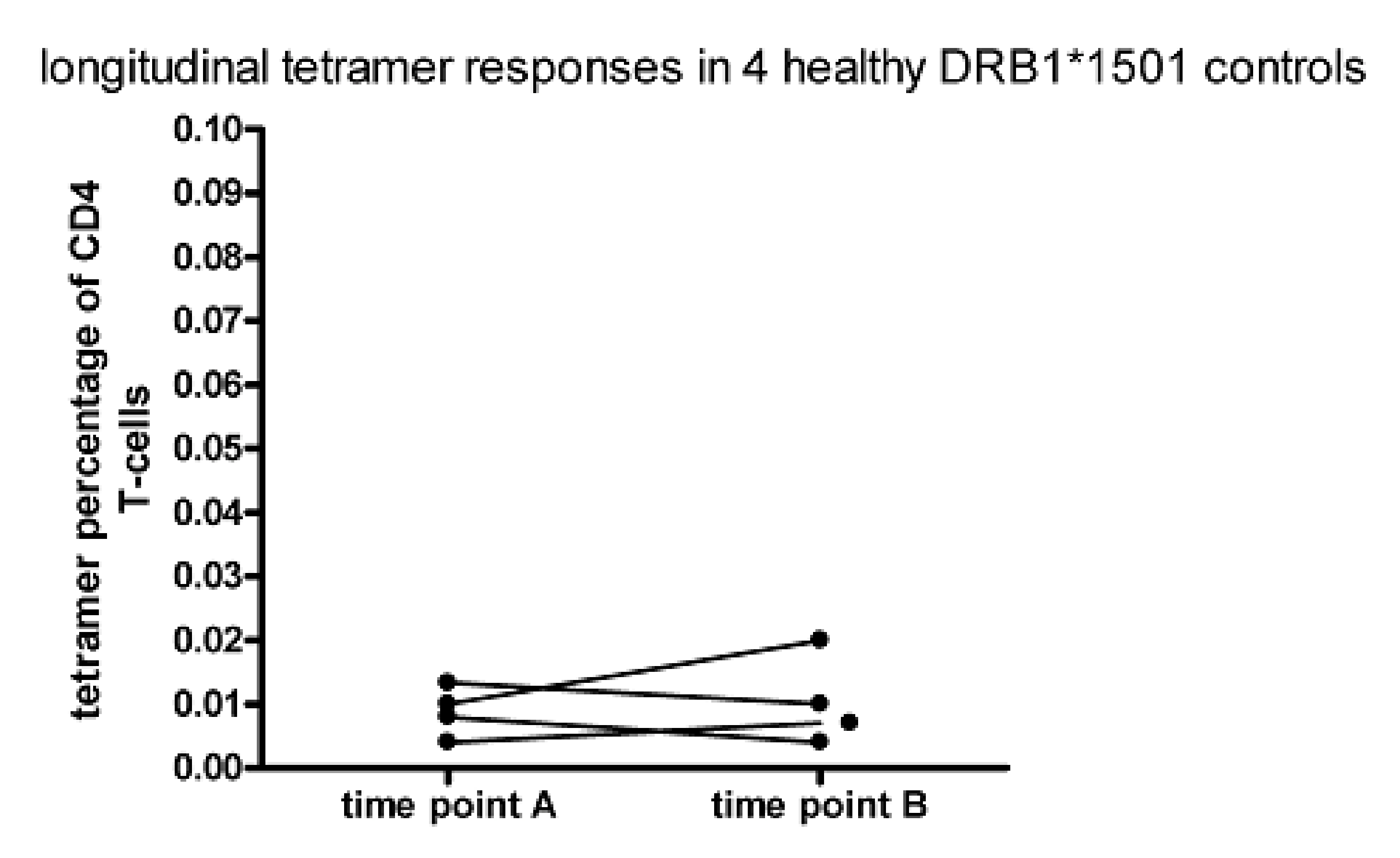

Supplement: Figure S5 — Frequency of tetramer specific T-cells was enumerated by flow cytometry in 4 HLA DRB1*1501-positive non-wasp allergic individuals who did not undergo immunotherapy at time point A and timepoint B (4–6 weeks later). There was no significant difference in the frequency of tetramer binding T-cells, p = 0.69, student's paired t-test. (0.25 MB TIF) [file pone.0011028.s005.tif]

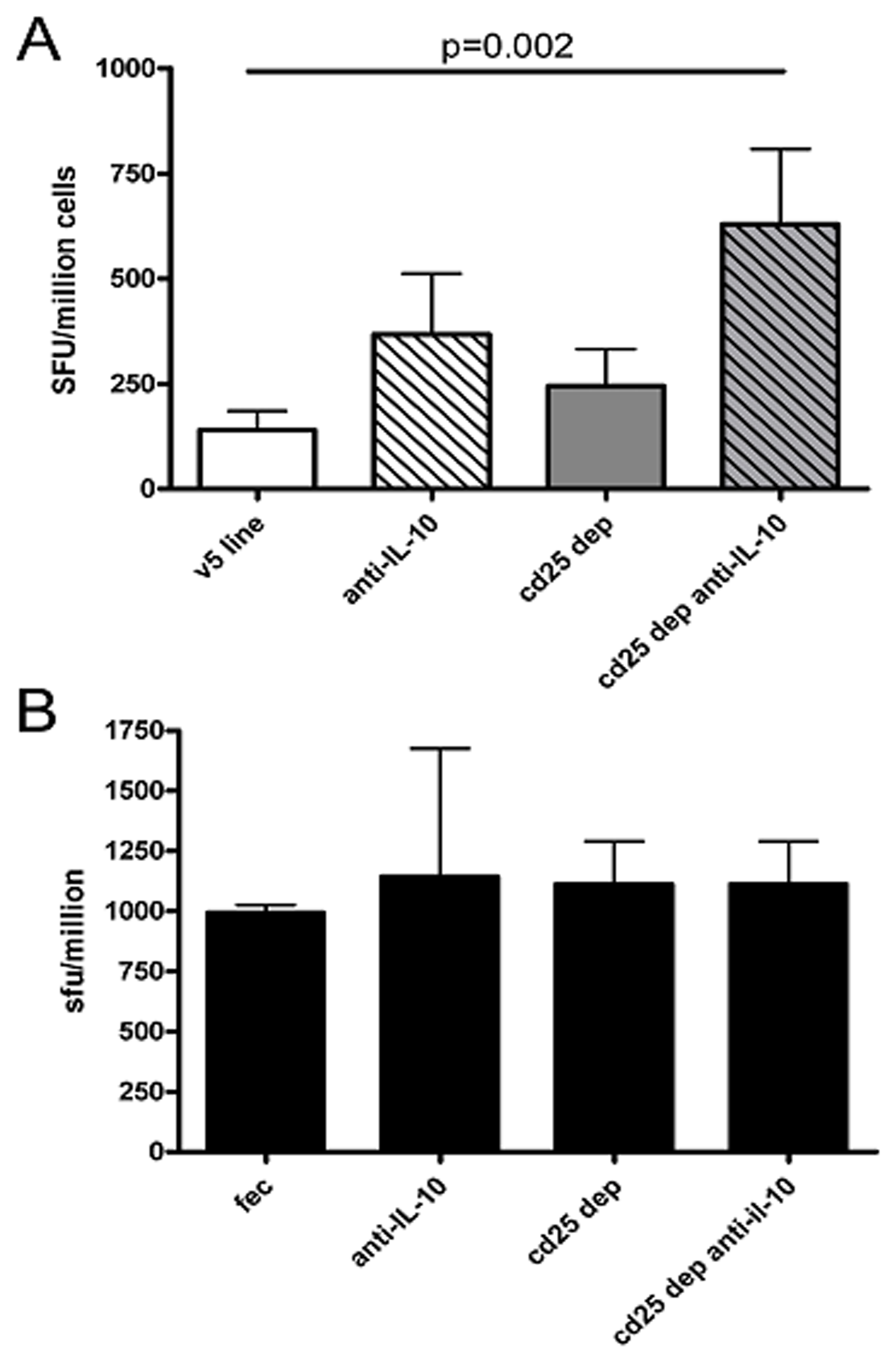

Supplement: Figure S6 — (A) Short term T-cell lines to rVes v 5 were generated in 4 wasp allergic individuals at week 12 of immunotherapy in the presence of anti-IL-10 (diagonal stripes) and after magnetic bead depletion of CD25hi T-regs(filled). T-cell lines were then stimulated overnight with Ves v 5 peptide pools (10 µM) and IL-4 responses were detected by ELISpot. The results are expressed as means and SD spots per million cells. (B) Short term lines were also generated to FEC peptide pool with anti-IL10 and CD25 depletion and re-stimulated overnight with FEC peptide pools on IFN-γ ELISpot plates. Mean and SD spot forming units per million cells are shown. (0.88 MB TIF) [file pone.0011028.s006.tif]
